# Supplementary figures and images for: E6 proteins from high-risk HPV, low-risk HPV, and animal papillomaviruses activate the Wnt/β-catenin pathway through E6AP-dependent degradation of NHERF1
Source: PLoS Pathog. 2019 Apr 19;15(4):e1007575. doi: 10.1371/journal.ppat.1007575 (PMC6493770; doi:10.1371/journal.ppat.1007575)

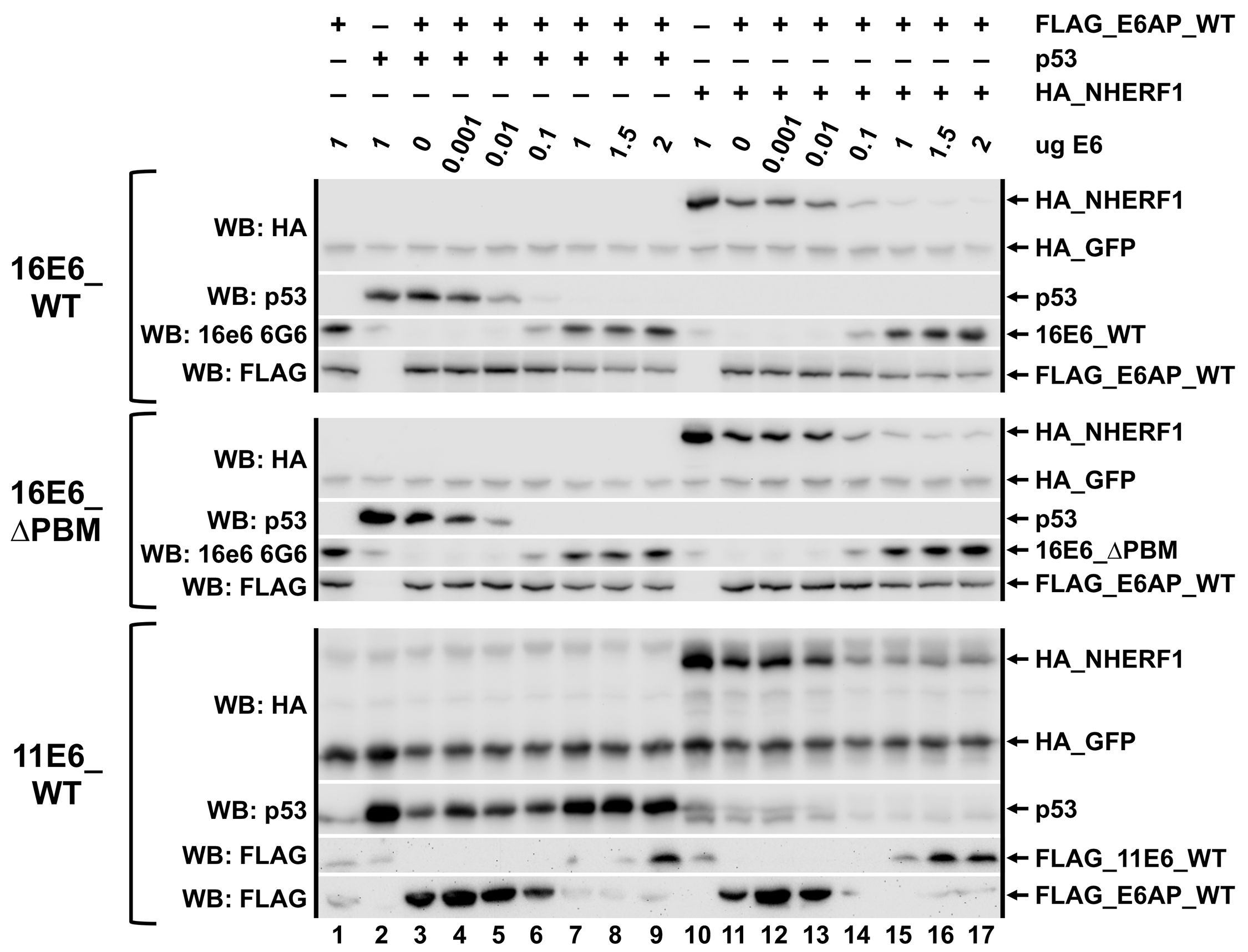

Supplement: S1 Fig — Titrations of the indicated three different E6 proteins (16E6_WT, 16E6_ΔPBM, and 11E6_WT) were co-transfected with FLAG_E6AP_WT (1 ug), HA_GFP (0.02 ug), and either HA_NHERF1 (0.5 ug) or p53 (0.5 ug) in E6AP-null 8B9 cells. A representative blot of the triplicate experiments for each E6 protein is shown. Increased E6 expression for 16E6_WT, 16E6ΔPBM, and 11E6_WT resulted in decreased NHERF1 protein levels. Both 16E6_WT and 16E6ΔPBM degrade p53 with increasing E6 expression. Overexpression of 11E6 _WT (>0.1 ug E6) resulted in degradation of co-expressed E6AP_WT. (TIF) [file ppat.1007575.s001.tif]

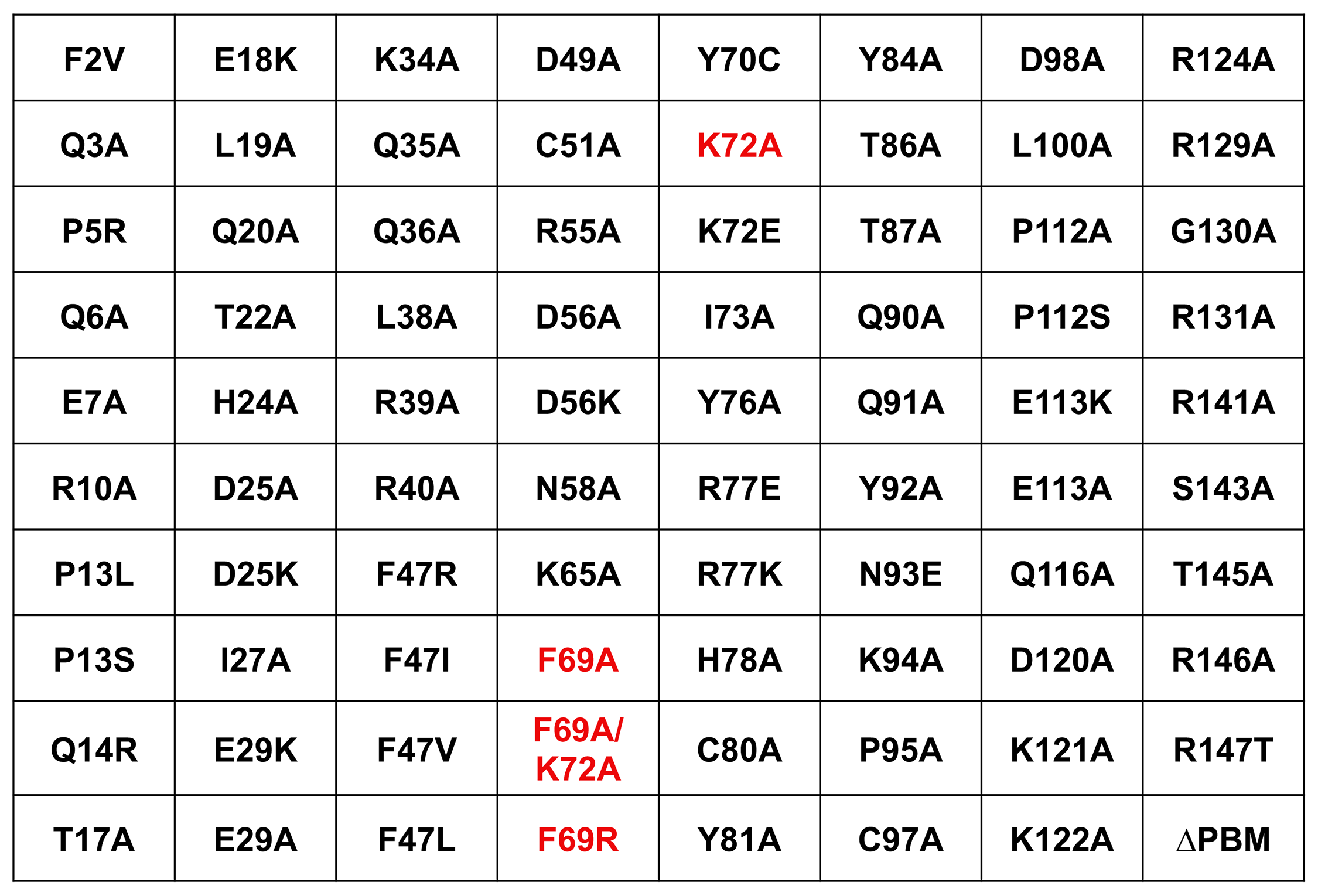

Supplement: S2 Fig — The 16E6 crystal structure (PDB file 4GIZ) was examined for residues that were at least 20% exposed as determined by the Swiss PDB Viewer. Point mutants of these identified amino acids were then screened to identify which residue(s) resulted in an E6 protein that was selectively defective for degrading NHERF1 but retained degradation of p53. Residues of interest are indicated in red. (TIF) [file ppat.1007575.s002.tif]

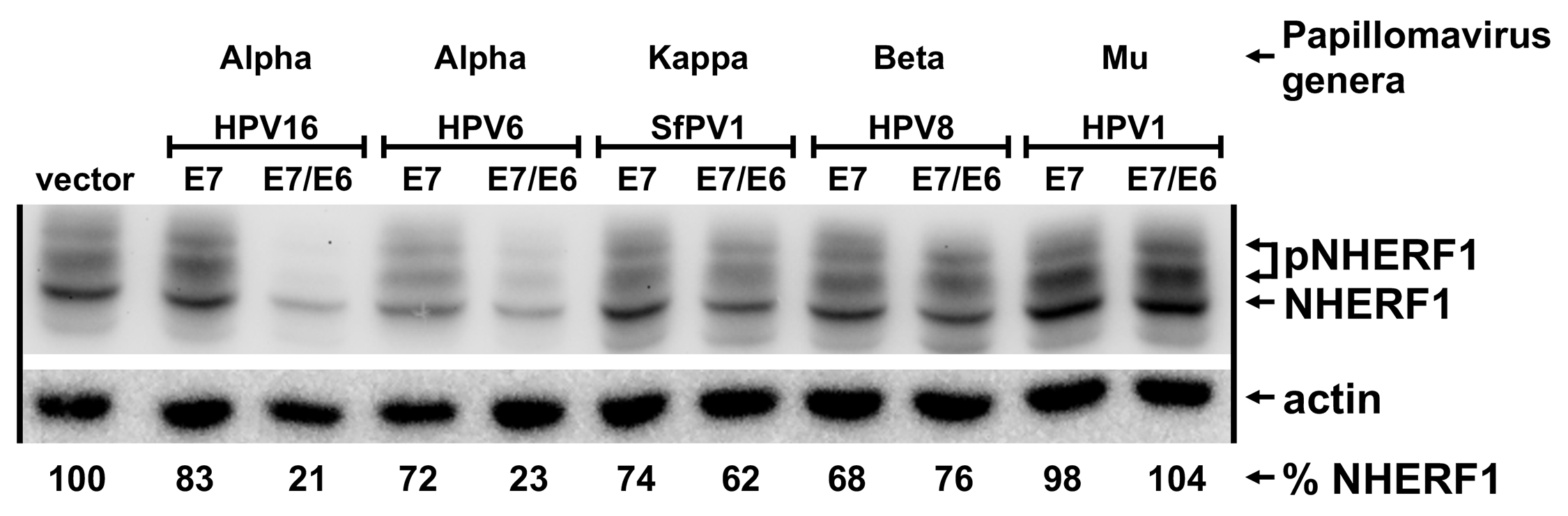

Supplement: S3 Fig — Keratinocytes retrovirally transduced with vector or the indicated E7 and/or E6 proteins were seeded at equal confluency. Levels of endogenous NHERF1 were determined by western blot. Levels of phosphorylated NHERF1 (pNHERF1) were unchanged in keratinocytes expressing empty vector compared to the various E7 proteins. Keratinocytes expressing the E6 protein from high-risk (HPV16) and low-risk (HPV11) degraded NHERF1. H = Homo sapiens (human), Sf = Sylvilagus floridanus (Cottontail rabbit; CRPV1). Caption credit: Accardi R, Rubino, R, Scalise M, Gheit T, Shahzad N, Thomas M, Banks L, Indiveri C, Sylla BS, Cardone RA, Reshkin SJ, Tommasino M, J Virol 85:3027–3030, 2011, 10.1128/JVI.00114-11. (TIF) [file ppat.1007575.s003.tif]

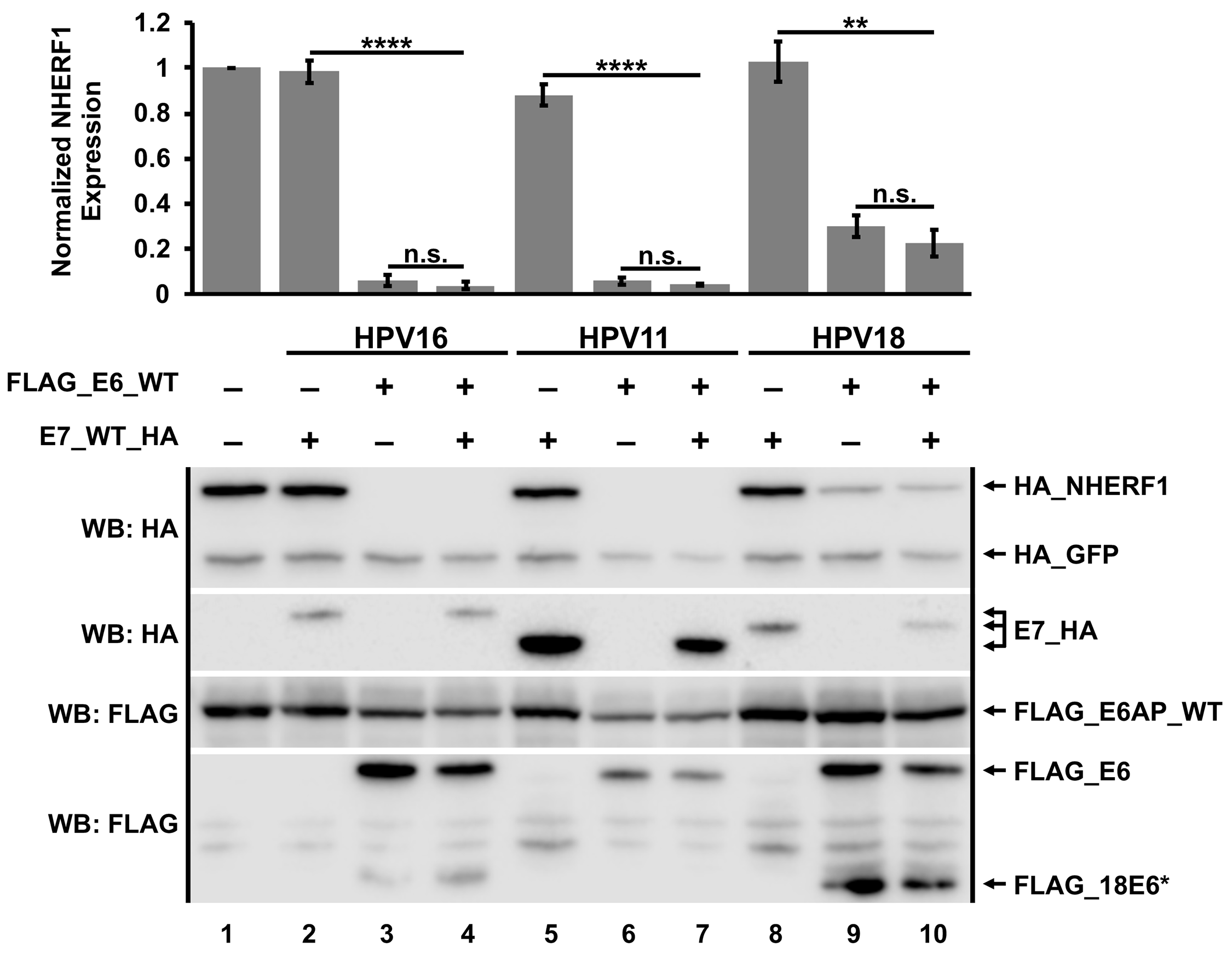

Supplement: S4 Fig — C33A cells were co-transfected with the following plasmids: HA_NHERF1 (0.4 ug), FLAG_E6AP_WT (0.35 ug), HA_GFP (0.08 ug), the indicated E6 protein (0.3 ug), and the indicated E7 protein (0.3 ug). HA_NHERF1 levels were determined by western blot. FLAG_18E6* is a truncated splice isoform of 18E6. Quantitation is derived from three experimental replicates. A representative blot and means of triplicate independent experiments ± standard error are shown. N = 3. **<0.01, ***<0.001, ****0.0001, n.s. = no significance by Student’s t-test. (TIF) [file ppat.1007575.s004.tif]
